# Structural Basis of Ets1 Cooperative Binding to Widely Separated Sites on Promoter DNA

**DOI:** 10.1371/journal.pone.0033698

**Published:** 2012-03-14

**Authors:** Nigar D. Babayeva, Oxana I. Baranovskaya, Tahir H. Tahirov

**Affiliations:** Eppley Institute for Research in Cancer and Allied Diseases, University of Nebraska Medical Center, Omaha, Nebraska, United States of America; Saint Louis University, United States of America

## Abstract

Ets1 is a member of the Ets family of transcription factors. Ets1 is expressed in autoinhibited form and its DNA binding depends on partner proteins bound to adjacent sequences or the relative positioning of a second Ets-binding site (EBS). The autoinhibition of Ets1 is mediated by structural coupling of regions flanking the DNA-binding domain. The NMR structure of Ets1 revealed that the inhibitory regions comprised of helices HI1 and HI2 and H4 are packed together on the Ets domain to form an inhibitory module. The crystal structure of Ets1 unexpectedly revealed a homodimer in which homodimerisation occurs via swapping of HI1 helices. Modeling of DNA binding indicates that the Ets1 dimer can bind to two antiparallel pieces of DNA. To verify this, we crystallized and solved the structure of the complex comprised of Ets1 dimer and two pieces of DNA. DNA binding by Ets1 dimer resulted in formation of additional intermolecular protein•DNA interactions, implying that the complex formation is cooperative.

## Introduction

Ets1, a founding member of the Ets (E-twenty-six-specific) family of transcription factors, was initially identified as the protooncogene corresponding to v-*ets* of the E26 leukemia virus [1], [2]. Ets1 regulates expression of lymphocyte-specific genes [3], bone-specific genes [4], and genes involved in vascular development and angiogenesis [5]. Ets1 is amplified and rearranged in leukemia and lymphoma [6]. Elevated Ets1 expression has been observed in many invasive and metastatic solid tumors, including breast, lung, colon, pancreatic and thyroid cancer [6].

Ets family members contain a highly conserved DNA-binding Ets domain, an 85-amino acid winged helix-turn-helix DNA-binding domain which recognizes a core motif 5′-GGA(A/T)-3′ referred to as Ets-binding site (EBS) [7], [8]. The Ets proteins are often expressed in autoinhibited form and their DNA binding depends on partner proteins bound to adjacent sequences [9], [10], [11], [12], including the relative positioning of a second EBS [13]. In the case of Ets1, autoinhibition is mediated by structural coupling of the regions flanking the DNA-binding domain. The NMR structure of the partially inhibited Ets1 fragment aa 301–441 shows that the inhibitory regions, which are folded as helices HI1 and HI2 N-terminal to the ETS domain and H4 C-terminal to the ETS domain, are packed together on the Ets domain to form an inhibitory module [14]. Deletion of either region or disruption of the inhibitory module by point mutations of Ets1 resulted in 10- to 20-fold increases in DNA-binding affinity [15], [16], [17], [18], [19]. Ets1 autoinhibition is counteracted by direct interaction of the DNA-binding domain and/or autoinhibitory regions with regulatory partners, including Pax5 [11], Runx1 [20], [21], [22], [23], [24] and Runx2 [25], [26], or by DNA-mediated homodimerization [27], [28], [29]. In the latter case, two Ets1 molecules were found to bind cooperatively to the palindromic sequences in which two head-to-head EBS were separated by four base pairs [27], [28], [29], [30], [31], [32], [33], [34]. According to earlier studies, it is expected that upon DNA binding the inhibitory module of Ets1 is disrupted and the helix HI1 becomes disordered [35]. However, our study of Ets1 binding to palindromic EBS on stromelysin-1 promoter revealed that the structural integrity of the inhibitory module and its involvement in intermolecular interactions are essential for DNA-mediated homodimerization of Ets1 [36]. Because the regions flanking the Ets domain appear to fulfill dual and opposing roles such as autoinhibition and cooperative DNA binding, we revisited the interpretation of the role of Ets1 dimer formation which we observed in crystals (Tahirov, Inoue-Bungo and Ogata, PDB code 1gvj). Ets1 dimer with a similar overall conformation was observed in a different crystal form obtained in the Wolberger laboratory (PDB code 1mdo), to which they referred as a domain-swapped dimer [35]. Because Ets1 dimer retained its shape under different crystallization and crystal packing conditions, we looked at whether the dimerization may have any role in DNA binding. Modeling of DNA binding indicated that Ets1 dimer can bind to two antiparallel pieces of DNA. To verify Ets1 dimer binding to two separate pieces of DNA we crystallized and solved the structure of the complex comprised of Ets1 dimer and two pieces of DNA [referred to as (Ets1)_2_•2DNA]. The structure revealed that in spite of DNA binding the overall conformation of Ets1 dimer, including HI1 and HI2, remains the same. These data shows that at high local concentration Ets1 homodimerization and cooperative DNA binding may have a regulatory role.

## Results

### Overall structure

**Figure 1:**
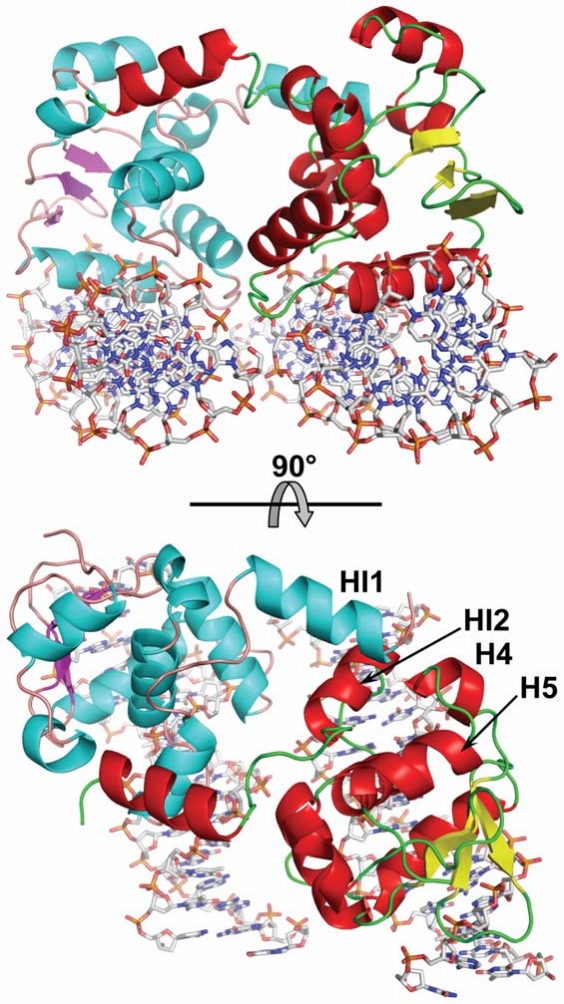
Overall structure of (Ets1)_2_•2DNA. Two orthogonal views are shown. Ets1 molecules are drawn as cartoons and DNA molecules are drawn as sticks. The helices, strands and coils are shown in cyan, magenta and light brown colors in one Ets1 molecule and respectively in red, yellow and green in another Ets1 molecule. The DNA molecules are colored by types of atoms: oxygen is red, nitrogen is blue, carbon is grey and phosphorus is orange. The labeled helices HI2, H4 and H5 are involved in docking of HI1 helix from another Ets1 subunit.

Crystals belong to the monoclinic space group P2_1_ and diffract up to 3.1 Å resolution. The structure was solved by the molecular replacement method and refined to an R_free_ of 28.4%. The asymmetric unit contains two Ets1 molecules (residues 280–441) forming a dimer. The electron density is absent for the amino acid residues 280–301 and 438–441 of each Ets1 and these residues are excluded from the model. The DNA-binding areas of each Ets1 subunit are on the same side of the dimer and docked on two antiparallel pieces of dsDNA from TCRα promoter. Each Ets1 is bound to a separate piece of dsDNA resulting in formation of (Ets1)_2_•2DNA quaternary complex (Fig. 1).

### Intermolecular interactions

Within the (Ets1)_2_•2DNA complex the Ets1•Ets1 interactions are observed at two equivalent positions (Fig. 1). They are similar to the intermolecular interactions found in the crystal structure of Ets1 dimer [35]. Briefly, at each position the interactions involve the N-terminal portion of HI1 helix from one subunit and H4 helix, a loop H4H5, HI2 helix and a loop HI1HI2 from another subunit (Fig. 1). The HI1 packs against the C-terminal of H4, making two main-chain to main-chain hydrogen bonds. The hydrogen bonds are also observed between the side chains of Lys305 and Tyr424 and between the carbonyl oxygen of Gly302 and hydroxyl oxygen of Tyr329. The small hydrophobic core is formed by packing the side chains of Phe304 and Tyr307 against a hydrophobic surface formed by Ile321, Pro322, Ala325, Leu326, Tyr329, Leu421 and Leu422.

**Figure 2:**
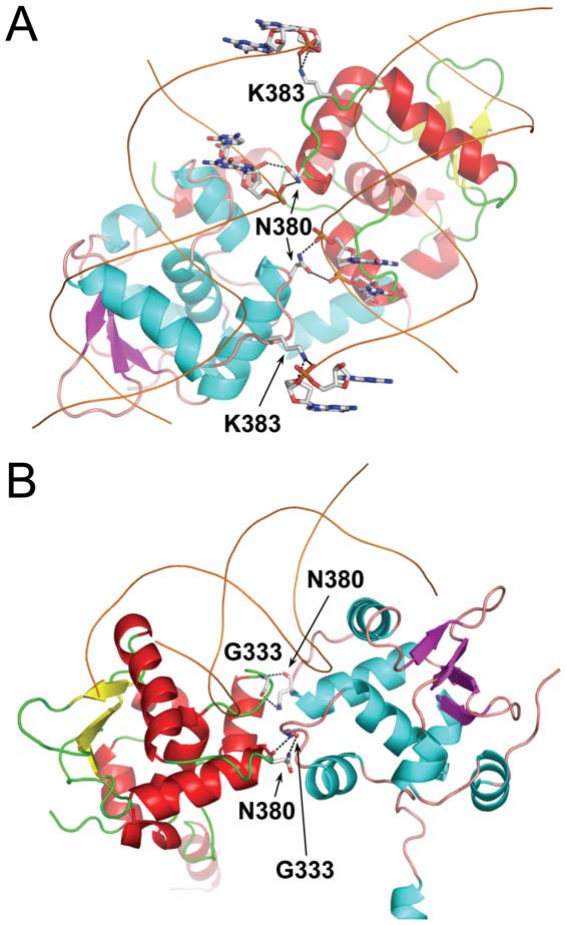
Comparison of intermolecular hydrogen bonds in (Ets1)_2_•2DNA and (Ets1)_2_•DNA complexes. (**A**) Intermolecular hydrogen bonds between Ets1 and the neighboring DNA duplex within (Ets1)_2_•2DNA complex. (**B**) Intermolecular hydrogen bonds between Ets1 subunits in (Ets1)_2_•DNA complex. In panels **A** and **B** Ets1 and DNA molecules are drawn as cartoons and interacting residues are drawn as sticks and labeled. The potential hydrogen bonds are shown as dotted lines. The color codes are as in Figure 1.

**Figure 3:**
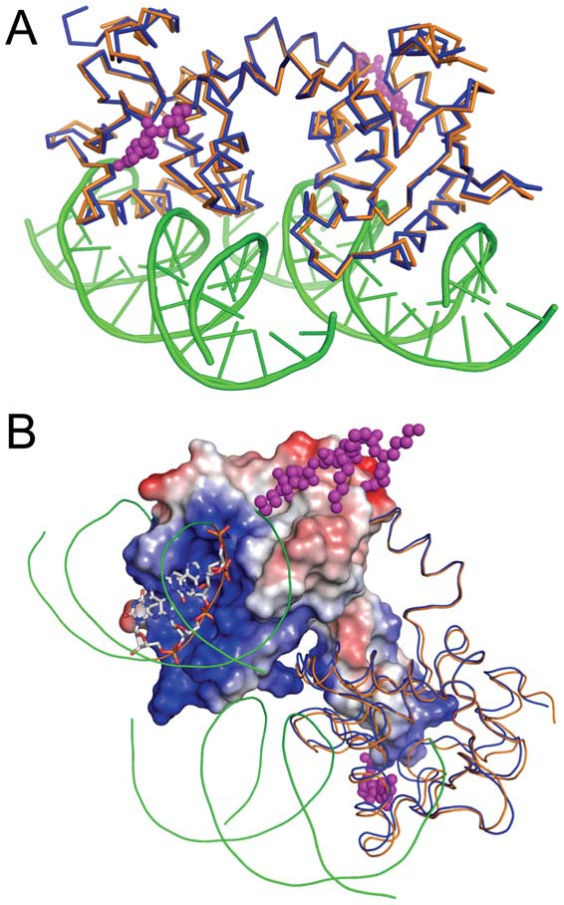
Comparison of Ets1 dimers with and without DNA. (**A**) Superimposition of DNA-free Ets1 dimer (blue) (PDB access code 1gvj) with DNA-bound Ets1 dimer (orange). (**B**) The molecules are superimposed and colored as in **A**. The charged surface is drawn for one of the Ets1 subunits in (Ets1)_2_•2DNA complex and the DNA residues docked to the surface of Ets1 are shown as sticks. The positively and negatively charged surface areas of Ets1 are in blue and red, respectively. In panels **A** and **B** the magenta balls highlight the atoms of the N-terminal residues of DNA-free Ets1 that are extended toward the DNA-binding surface of Ets1.

The DNA binding by each Ets1 subunit is similar to that reported for Ets1•DNA complex [11]. However, within (Ets1)_2_•2DNA complex additional hydrogen bonds are observed between each Ets1 subunit and the neighboring DNA duplex. The potential hydrogen bonds involve the side chains of Asn380 and Lys383 from the loop H2H3 and DNA phosphate oxygen (Fig. 2). The positively charged surface of Ets1 facing the neighboring DNA (Fig. 3) also contributes to the overall stability of (Ets1)_2_•2DNA complex since the long-range electrostatic interactions enhance the DNA-binding affinity of the protein [37].

### Comparison with DNA-free Ets1 dimer

Superimposition of Ets1 dimers with and without DNA shows that DNA binding introduces only minor local changes in Ets1 dimer structure (Fig. 3A) with the root-mean-square deviations for the 271 matched α-carbon atoms at 0.86 Å. The only notable difference is the disorder of the seven N-terminal residues within (Ets1)_2_•2DNA complex. In DNA-free Ets1 dimer the N-terminal residues are extended toward the protein's DNA backbone-binding surface and form two additional intermolecular hydrogen bonds at each site, His298 ND1…Ser 420 O and Lys299 NZ…Tyr329 O. The direction of the residues N-terminal to HI1 indicates that the extended N-terminal would mask the DNA backbone-binding surface of Ets1 (Fig. 3B). This is consistent with the autoinhibitory role of the residues N-terminal to HI1.

### Comparison with cooperative binding to palindromic EBS

Structural studies of two Ets1 bound to palindromic EBS on stromelysin-1 promoter [further referred to as (Ets1)_2_•DNA complex] revealed two areas of intermolecular interactions that are essential for cooperative DNA binding, and both areas contributed to the stability of the inhibitory module [36]. Interestingly, the loop H2H3 harboring Asn380 and Lys383 appears to play an important role in intermolecular interactions in both the (Ets1)_2_•2DNA and (Ets1)_2_•DNA complex structures. In the former structure it interacts with the backbone of DNA that is docked to the second Ets1 subunit (Fig. 2A), and in the latter structure it interacts with loop HI2H1 of the second subunit (Fig. 2B). In the case of (Ets1)_2_•DNA complex the mutation of Asn380 to alanine resulted in the loss of cooperative DNA binding and in a reduction of activity at stimulating the stromelysin-1 promoter.

### Inhibitory module

**Figure 4:**
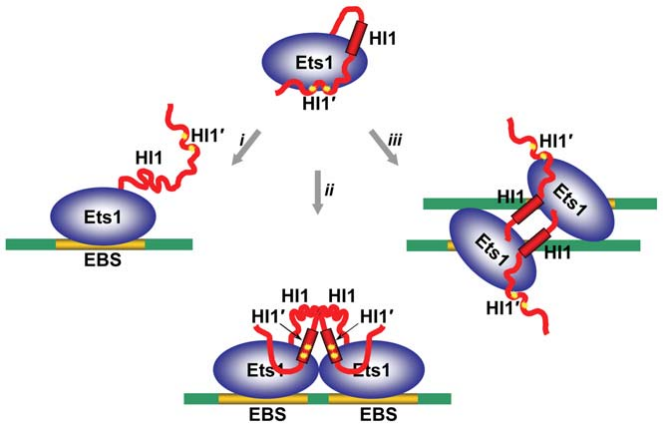
Mechanisms of releasing the Ets1 autoinhibition upon DNA binding. Three possible models of counteracting the autoinhibition: (*i*) by disruption of autoinhibitory helix HI1 interactions and its disorder upon binding to a single EBS [35], (*ii*) by replacement of helix HI1 by induced helix HI1′ upon binding to a palindromic EBS [36], (*iii*) and by direct competition with autoinhibitory sequences upon binding to widely separated EBSs (this report).

Another common feature of Ets1 dimers found in crystal structures, including Ets1 dimer, (Ets1)_2_•DNA and (Ets1)_2_•2DNA, is conservation of the structure of inhibitory module comprised of helices HI1, HI2 and H4, and the loops HI1HI2 and H4H5 (Fig. 1). Unlike these structures, DNA binding by monomeric Ets1 alone or in complex with a regulatory partner results in the disorder of helix HI1 [11], [35]. Together, the DNA-free and DNA-bound crystal structures of Ets1 indicate that Ets1 autoinhibition could be counteracted by at least three different mechanisms: by disruption and disorder of autoinhibitory helix HI1 [35], by replacement of helix HI1 [36], and by direct competition with autoinhibitory sequences (Fig. 4). In all cases the end result is unmasking the DNA-binding surface of Ets1.

## Discussion

Interactions between transcriptional factors are often weak and transient, and are physiologically relevant only at high concentrations. It is possible that the weaker interactions prevent them from accidental aggregation. However, binding to adjacent sites on promoters and enhancers increases the local concentration of transcriptional factors dramatically, even if their concentration in the cell is very low [38]. An increase in local concentration also occurs for factors bound to widely separated sites on a promoter due to looping of promoter DNA [38], [39], [40]. Observation of the identical dimerization mode of Ets1 in crystals obtained under different conditions, with different truncated Ets1 constructs, and having different crystal packing, once again pointed to a tendency of Ets1 for dimerization at high concentrations. The Ets1 homodimer observed in the crystals might also form at a high local concentration of Ets1. Such a high local concentration would be achieved only if the Ets1 molecules bind to adjacent sites on DNA like in stromelysin-1 promoter [36] or to sites that are widely separated in sequence but closely positioned in space because of DNA looping. To test this hypothesis, we crystallized and solved the crystal structure of Ets1 homodimer bound to two separate dsDNA fragments. The structure revealed that Ets1 homodimer binds to parallel pieces of dsDNA having EBS with opposite orientation. The structure also revealed that Ets1 homodimer readily recognizes two antiparallel pieces of dsDNA without changing the conformation of DNA-binding domains and inhibitory helices.

**Figure 5:**
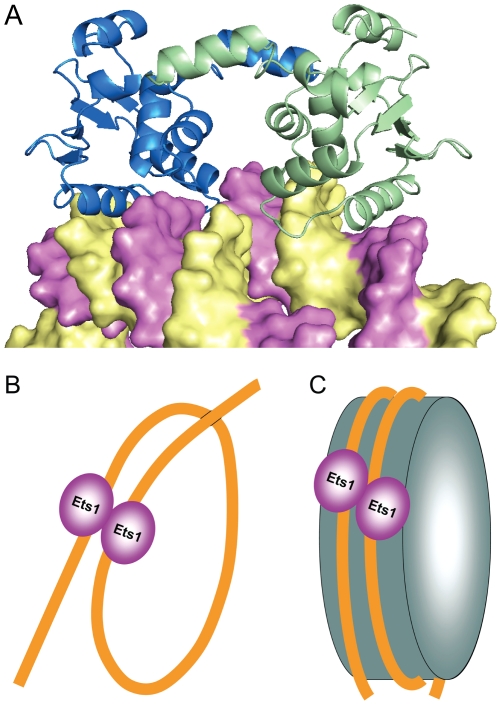
Models of Ets1 binding to widely separated EBS. (**A**) Docking of Ets1 homodimer to nucleosomal DNA based on the superimposition of DNA in the (Ets1)_2_•2DNA structure and the high-resolution structure of a nucleosome core particle (PDB access code 1kx5) [49]. Ets1 molecules are displayed as blue and green cartoons and DNA is displayed as a surface with the strands highlighted in yellow and magenta colors. (**B**) and (**C**) Schematic representation of two models of Ets1 cooperative binding to widely separated EBS on promoter DNA: (**B**) binding via looping of promoter DNA and (**C**) binding to a nucleosome core particle.

It is not known whether Ets1 homodimer alone can support DNA looping or whether the function of other DNA-bending factors is necessary. However, Ets1 was shown to be capable of binding to nucleosomal DNA with the same order of affinity as that of binding to naked DNA [41]. Among the well characterized examples are Ets1 binding to nucleosomal DNA of HIV-1 long terminal repeat [42], platelet factor 4 [41] and immunoglobulin μ heavy chain [43] enhancers. That is why it is interesting whether Ets1 is predisposed for binding to nucleosomal DNA also as a homodimer. Indeed, the separation of DNA fragments and exposed major groove positions of nucleosomal DNA coincide with Ets1-binding sites in (Ets1)_2_•2DNA. Figure 5A shows that Ets1 homodimer could be readily docked to nucleosomal DNA with only minor adjustments in Ets1 homodimer and nucleosomal DNA structures required for a tight complex formation.

Finally, we speculate that based on the (Ets1)_2_•2DNA complex structure two models of Ets1 cooperative binding to widely separated EBS are possible: cooperation via DNA looping (Fig. 5B) and cooperation on nucleosome core particles (Fig. 5C). Such cooperative binding would give an advantage to Ets1 for competing with other Ets family members both by dimerization and by making additional interactions with backbone phosphates and bases of DNA fragments harboring a second subunit of Ets1 dimer.

## Materials and Methods

### Preparation of (Ets1)_2_•2DNA

Ets1_280–441_ has been cloned, expressed and purified according to reported protocols [36]. A double-strand oligonucleotide containing Runx1 and Ets1-binding region of TCR-α promoter was prepared by annealing synthetic oligonucleotides 5′-GGAAGCCACATCCTCT-3′ and 5′-CAGAGGATGTGGCTTC-3′ synthesized by the Eppley Molecular Core laboratory of University of Nebraska Medical Center. Each oligonucleotide was dissolved in 10 mM Tris•HCl (pH 7.5), 1 mM EDTA and 1 M NaCl at a concentration of 0.2 mM. Oligonucleotide pairs were annealed by heating to 95°C for 5 min and gradually cooling to room temperature over 3 h by using a PCR thermal cycler. The annealed DNA was desalted, dried and dissolved in 10 mM Tris•HCl (pH 8.0). Ets1_280–441_ and the TCRα dsDNA were mixed in 1∶1.05 ratio, incubated at room temperature for 20 min in 5 m*M* Tris•HCl buffer with pH 7.5 and 5 m*M* DTT and concentrated to 8.5 mg•ml^−1^. Complex formation was monitored by electrophoresis to confirm that the excess of DNA is approximately 5%. The complex containing solutions were stored in small aliquots at 253 K and each aliquot thawed only once before crystallization.

### Crystallization of (Ets1)_2_•2DNA and diffraction data collection

Crystallization screening was performed using Natrix screen kit (Hampton Research) by the sitting-drop vapor-diffusion method at 295 K by mixing 1 µl protein•DNA solution with 1 µl reservoir solution. The rectangular thin plate crystals appeared in the 47^th^ and 48^th^ conditions of the Natrix screen kit. The optimizations of crystal growth conditions were performed with variation of additives, polyethylene glycols and the concentration of components. Thicker diffraction-quality plate crystals growing in aggregates were obtained at 295 K in 200 m*M* ammonium chloride, 10 m*M* calcium chloride, 50 m*M* Tris-HCl buffer (pH 8.5), 21% v/v polyethylene glycol monomethyl ether 2000 (PEG MME 2000) and 3% v/v glycerol. The best-shaped crystals were surgically separated from aggregates using microtools, washed four times and used for macroseeding in the drops of mother liquor equilibrated against the reservoir solution containing 18.5% v/v of PEG MME 2000 instead of 21% v/v for 1 day. For X-ray diffraction data collection the crystals were soaked in cryoprotectant and mounted in nylon-fiber loops and flash-cooled in a dry nitrogen stream at 100 K. Cryoprotectant was prepared by the addition 12% v/v of PEG 400 to a reservoir solution. Preliminary X-ray examinations of crystals were carried out using Rigaku R-AXIS IV imaging plate with Osmic VariMax™ HR mirror-focused Cu K_α_ radiation from Rigaku FR-E rotating-anode generator operated at 45 kV and 45 mA. The final data set was collected on Argonne National Laboratory Advanced Photon Source beamline 24ID-C using an ADSC Q315 detector. All intensity data were indexed, integrated and scaled with DENZO and SCALEPACK from the HKL2000 program package [44]. The crystals belong to the monoclinic space group *P*2_1_ and diffract up to 2.8 Å resolution; however, the diffraction beyond 3.1 Å is anisotropic and the spots are too wide and elongated. The crystal parameters and data-processing statistics are summarized in Table 1. Unlike the (Ets1)_2_•2DNA crystals, the crystals of Ets1 dimer (PDB code 1gvj) were grown in the 9^th^ condition of Hampton Research Crystal Screen kit and belong to a triclinic *P*1 space group.

**Table 1:** Data collection and refinement statistics.

Data collection	
Space group	*P*2_1_
Cell dimensions:	
a (Å)	50.18
b (Å)	98.12
c (Å)	53.58
β (°)	109.67
Resolution (Å)*	30-3.0 (3.11-3)
Unique reflections	9369
*R*_merge_ (%)*	8.0 (36.3)
*I*/σ(*I*)	12 (2.2)
Completeness (%)	94.7 (87.9)
Redundancy	2.6 (1.9)
Temperature (K)	100
Mosaicity (°)	0.7–1.3

Refinement	
Resolution (Å)	30-3.1
No. reflections	8427
*R*_work_/*R*_free_	0.23/0.284
No. atoms/B-factors (Å^2^)	
Protein	2236/54.0
DNA	1300/43.3
Water	33/21.7
R.m.s. deviations	
Bond lengths (Å)	0.008
Bond angles (°)	1.3
Ramachandran plot	
Favored (%)	87.0
Allowed (%)	12.2
Generously allowed (%)	0.8

* Values in parentheses are for the last shell.

### (Ets1)_2_•2DNA structure determination

The structure was determined by the molecular replacement method starting with the coordinates of Ets1 (PDB entry 1gvj with an R_cryst_ of 20.8% and an R_free_ of 23.5% at 1.53 Å resolution). The asymmetric unit contained Ets1 homodimer and two pieces of dsDNA. The major manual rebuilding of the initial model was performed with TURBO-FRODO software. The refinement at 3 Å resolution resulted in a significant 25% jump of R-free for the reflections in the 3.1–3.0 Å shell. That is why the model was refined at 3.1 Å resolution to an R_cryst_ of 22.5% and an R_free_ of 28.3%. CNS version 1.1 [45] was used for all crystallographic computing. Application of zonal scaling [46] and bulk solvent correction improved the quality of electron density maps. The final refinement statistics are provided in Table 1. The figures containing molecular structures were drawn with PyMOL. The electrostatic surface potential was calculated and displayed with PyMOL [47]. Similar surface charge distribution was obtained also with GRASP [48].

### Accession numbers

Atomic coordinates and structure factors of (Ets1)_2_•2DNA have been deposited in the Protein Data Bank with accession number 3ri4.
